# *TaMFT-A1* Is Associated with Seed Germination Sensitive to Temperature in Winter Wheat

**DOI:** 10.1371/journal.pone.0073330

**Published:** 2013-09-12

**Authors:** Lei Lei, Xinkai Zhu, Shuwen Wang, Meirong Zhu, Brett F. Carver, Liuling Yan

**Affiliations:** 1 Department of Plant and Soil Sciences, Oklahoma State University, Stillwater, Oklahoma, United States of America; 2 Key Laboratory of Crop Genetics and Physiology of Jiangsu Province, Yangzhou University, Jiangsu, China; Wuhan University, China

## Abstract

The ability of seed to germinate under favorable environmental conditions is critical for seedling emergence, plant establishment, subsequent development and growth of adult plants, and it is controlled by internal genetic factors and external environmental factors. Winter wheat in the southern Great Plains is often planted six weeks before the optimal planting date to produce more biomass for cattle grazing during the winter season. A high seed germination rate in this higher soil temperature environment is required for this specific management system. In this study, a major QTL for temperature-sensitive germination was mapped on the short arm of chromosome 3A (*QTsg.osu-3A*) in a RIL population generated from two winter wheat cultivars. Furthermore, *TaMFT-A1*, previously reported to regulate seed dormancy and pre-harvest sprouting in spring wheat cultivars, was mapped tightly associated with the peak of *QTsg.osu-3A*. However, allelic variation in *TaMFT-A1* between the two winter wheat cultivars differed from that was observed in spring wheat cultivars. There were 87 SNPs (single nucleotide polymorphisms) and 12 indels (insertions/deletions) in *TaMFT-A1* between the Jagger allele for high germination and the 2174 allele for low germination in the after-ripened seeds, in comparison with 2 SNPs between the two alleles for differential pre-harvest sprouting in spring wheat cultivars. The Jagger *TaMFT-A1* allele is a novel haplotype and appears extensively in winter wheat cultivars. *TaMFT-A1* transcript levels were up-regulated by high temperature but down-regulated by low temperature or seed storage time. These findings suggest that *TaMFT-A1* may invoke different mechanisms for controlling seed dormancy/germination among winter wheat cultivars.

## Introduction

Plant seed germinability, the ability of a viable seed to germinate under favorable environmental regimes, is critical to contribute to seedling performance important for plant establishment and subsequent development and growth of plants [1], [2], [3]. Seed germination is controlled by given internal factors, such as seed dormancy and hormones that promote or prevent germination through physiological mechanisms, and by external factors such as temperature, water, oxygen and light [4]. In plants, temperature is the most influential factor among external conditions controlling seed germination [5].

The responses of seeds to temperature are complex. The optimum temperature to gain maximum seed germinability is approximately 20–25°C. A low temperature (<4°C) can break seed dormancy and promote seed germination [6], whereas a high temperature (>35°C) has inhibitory effects on germination in wheat [7], [8]. Even after the dormancy is broken, the seeds may not be able to germinate under the high temperature, which confers an adaptive mechanism for plants to germinate with seasonal changes in different geographical areas [9], [10].

Winter wheat in the southern Great Plains is preferred to plant in the early September or six weeks before the optimal planting date to produce more wheat biomass as forage for cattle grazing during the following winter season [11]. A high germination ability of seed in the presence of high temperature is required to adapt to the specific agronomic management system. However, some cultivars can germinate at the optimum temperature but cannot germinate at a high temperature, which is a biological phenomenon called high temperature germination sensitivity. The high temperature germination sensitivity has been reported in other plants, such as thermoinhibition in Arabidopsis [12], or thermodormancy in oat [13] and in lettuce [14].

The molecular mechanism underlying high temperature germination sensitivity may vary by plant species. In Arabidopsis, *FLC* was recently linked to the regulation of temperature-dependent germination; seeds with high-temperature thermodormancy exhibited high *FLC* expression during germination at low temperature [15], [16]. In lettuce, a quantitative trait locus (QTL) *Htg6.1* for thermodormancy (a failure to germinate when imbibed at temperatures above 25–30°C) was identified associated with *LsNCED4*, a gene in the ABA biosynthetic pathway [14]. Mutants exhibiting altered ethylene synthesis or sensitivity have been identified that also exhibited germination tolerance at high temperatures. However, little is known about the genetic basis and molecular mechanism underlying temperature sensitivity germination in wheat.

Genetic approaches have identified several genes that affected seed dormancy and germination in wheat, but most of these studies have been focused on pre-harvest sprouting. More than 15 genetic loci have been reported to associate with this critical trait in a large range of wheat areas worldwide [17], and a QTL that appears to have stable and large effects on dormancy and germination is *QPhs.ocs-3A* on the short arm of chromosomes 3A [18]. In a recent study, *MFT-3A*, a homoeologue of *MOTHER OF FT* (Flowering locus T) and *TFL1* (Terminal Flower 1-like) genes, was mapped to chromosome 3A and associated with the seed dormancy at *QPhs.ocs-3A* in *T. aestivum*. *MFT-3A* is hereafter changed to *TaMFT-A1* to be consistent with wheat gene terminology. *TaMFT-A1* was mapped based on a single nucleotide polymorphism in its promoter region [2]. The up-regulation of the *TaMFT-A1* transcriptional level was associated with strong dormancy of seed in spring wheat cultivars.

Winter wheat and spring wheat may not share common biological mechanisms controlling seed germination, because their seeds are produced under different environment regimes [11]. Spring wheat seed usually has no or weak dormancy that results in immediate germination after harvest or even pre-harvest sprouting, whereas winter wheat usually has strong dormancy that prevents germination for the seed that has been stored for several months [19], [20], [21], [22]. The initial goal of this study aimed to identify genetic loci associated with the sensitivity of seed germination to temperature in winter wheat. After a major QTL for temperature-sensitive germination was mapped to the *TaMFT-A1* locus, this study was switched to analyze allelic variation in *TaMFT-A1* and regulation of its expression by temperature in winter wheat.

## Materials and Methods

### Plant Materials

Two winter wheat cultivars, ‘Jagger’ and ‘2174’, were observed a large variation in seed germination in our annual nurseries. Based on field observations, Jagger has high seed germination, whereas 2174 has low seed germination. Jagger and 2174 were originally used to generate a population of recombinant inbred lines (RILs) segregating for stem elongation and winter dormancy release in winter wheat [23]; a total of 350 SSR markers were mapped in this population [24]. The two parental lines and their RILs were tested for seed germination at different temperatures in growth chambers with temperature-, photoperiod-, and moisture-controlled conditions.

A total of 121 wheat accessions were genotyped using a new *TaMFT-A1* marker developed in this study, including 34 varieties released in the southern Great Plains in recent years [25], 19 pairs of parental lines that were used to construct mapping populations in the WheatCAP applied genomics project [25], 56 Chinese spring wheat cultivars that were genotyped for *VRN2* genes in a recent study [26], 3 diploid wheat accessions of *T. uratu* (2n = 2X = 14, AA), and 9 tetraploid wheat accessions of *T. turgidum* (2n = 4X = 28, AABB). Table S1 summarized genetic materials that were used in this study.

### Seed germination experiments

Seeds used for germination experiments were harvested from all RILs of the population grown in field in 2009 and 2010. After harvested, the seeds were stored at room temperature (20 to 22°C) till the date of experiments. Fifty intact seeds of each RIL or parent were evenly placed on a Petri dish with the lower half of a pre-wetted germination paper, and three replicates for each line were performed. The dishes were incubated in the dark at three temperature regimes: 1) low temperature at 4°C for 1 day and then constant temperature at 24°C, 2) the optimum temperature at constant 24°C, 3) high temperature at 35/27°C for day/night to simulate the seasonal temperatures at planting in the field. The germinated and un-germinated seeds were counted at 3, 5 and 7 days after planting. The criteria for germination were that either radical or shoot protruded out of seed coat.

### Isolation of the complete *TaMFT-A1* gene from winter wheat

Primers MFT-F1M (5′-GGCGCCGACATCGAGTTGTGG-3′) and MFT-A1R1 (5′-CATGCAAAGTGTGTGCGTATATATGTACC-3′) were used to amplify the complete *TaMFT-A1* gene from each of the Jagger and 2174 alleles, including 1 kb in the 5′ upstream from the start code for translation and 400 bp in the 3′ downstream from the stop code for translation, based on the sequences of Chinese Spring. Three nullisomic-tetrasomic (NT) lines, N3AT3B, N3BT3A, and N3DT3A, were used to determine specialty of the primers to chromosome 3A. PCR reactions were performed at 94°C for 3 minutes, following 40 cycles of 95°C for 30 seconds, 57°C for 30 seconds and 65°C for 4 minutes, with a final extension step at 65°C for 10 minutes. PCR products were run on a 1% agarose gel. The amplified fragments were purified using Gel/PCR DNA Fragment Extraction kit (IBI), fused to TA vector using a pGEM-T vector System I kit, and transformed to DH5^α^ competent cells. The plasmid DNA from a single colony was extracted and sequenced.

### Development of a PCR marker for *TaMFT-A1*

Two PCR markers were developed to map *TaMFT-A1*, which required the digestion of restriction enzymes or the distinguishing of a 3-bp indel between the alleles reported in previous studies [2]. In this study, a PCR marker was developed to map a 12-bp indel for variation between the Jagger and 2174 alleles. Primers MFT-A1F2 (5′-GAGCAAACATGTCCCGGTTCGTT-3′) and MFT-A1R2 (5′-ATCACCATGCACACACATACATAAATCACC-3′) were used to amplify partial *TaMFT-A1*, and the expected size was 331 bp for the Jagger allele and 319 bp for the 2174 allele. PCR was performed at 95°C for 3 minutes, following 40 cycles of 95°C for 30 seconds, 57°C for 30 seconds and 72°C for 1 minute, with a final extension step at 72°C for 10 minutes. PCR products were run on a 2% agarose gel.

### Mapping of *TaMFT-A1*

A genetic map was constructed from a set of 350 simple sequence repeat (SSR) markers previously used to map a major QTL for stripe rust associated with *Yr17* in the Jagger×2174 RIL population [24]. Each of genetic linkage groups was analyzed for QTLs controlling seed germination characterized in this study. The mean seed germination rate for each line from different environments was analyzed to determine significant effects of a single marker on resistance using one-way analysis of variance (ANOVA).

### Expression of *TaMFT-A1*

Primers QRT-MTF-F2 (5′-CCTCTACACCCTCGTGATGA-3′) and QRT-TaMTF-R6 (5′-GCACCACCACCTCACCTTTA-3′) were designed to test *TaMFT-1A* expression [2]. The pair primers were used to investigate transcriptional levels of *TaMFT-A1* in Jagger and 2174. Primers actin-F1 (5′-CTATGTTCCCGGGTATTGCT-3′) and actin-R1 (5′-AAGGGAGGCAAGAATCGAC-3′) were used to amplify transcripts of *actin* as endogenous control. The *TaMFT-A1* transcripts were assessed using the SYBR Green PCR Master Mix (Applied Biosystems, Life Technologies) and the Applied Biosystems 7500 Real-Time PCR Systems. Total RNA was extracted using Trizol regents (Invitrogen). RNA samples were treated with Deoxyribonuclease I and first-strand cDNA was synthesized using a SuperScript™ II Reverse Transcriptase kit (Invitrogen). qRT-PCR was performed using a 7500 Real-Time PCR System (Applied Biosystems) and iQ™ SYBR Green Supermix kit (BIO-RAD).

## Results

### Seed germination at the optimal temperature

**Figure 1:**
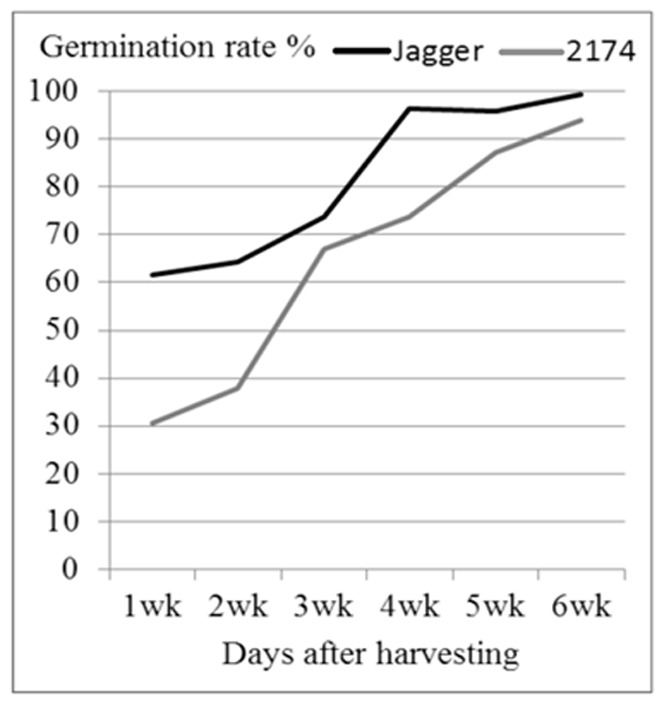
Germination rate of Jagger and 2714. The experiment was performed in 2011. Seed collected from a greenhouse was tested 1, 2, 3, 4, 5, and 6 weeks after harvesting in an incubator where constant temperature at 24°C was set up. The germination rate was an average of three replicates, and no standard error was calculated.

Both Jagger and 2174 are a winter wheat type. The mean germination rate at the optimal temperature (constant 25°C) was between 30% for 2174 and 60% for Jagger when their seeds were tested one week after harvested from a greenhouse (Fig. 1). When the time elapsed, the mean germination rate of the seeds stored at room temperature (20–25°C) was significantly increased, reaching approximately 90–95% at 6 weeks after harvest for both of the tested cultivars. These results indicated that the two winter wheat cultivars had some dormancy, and the dormancy of all the seeds had almost completely disappeared by 6 weeks after harvest. Jagger showed a significant higher germination rate than 2174; but the difference in seed germination between the two cultivars was decreased when the time elapsed (Fig. 1).

### A QTL for seed germination on chromosome 3A

**Figure 2:**
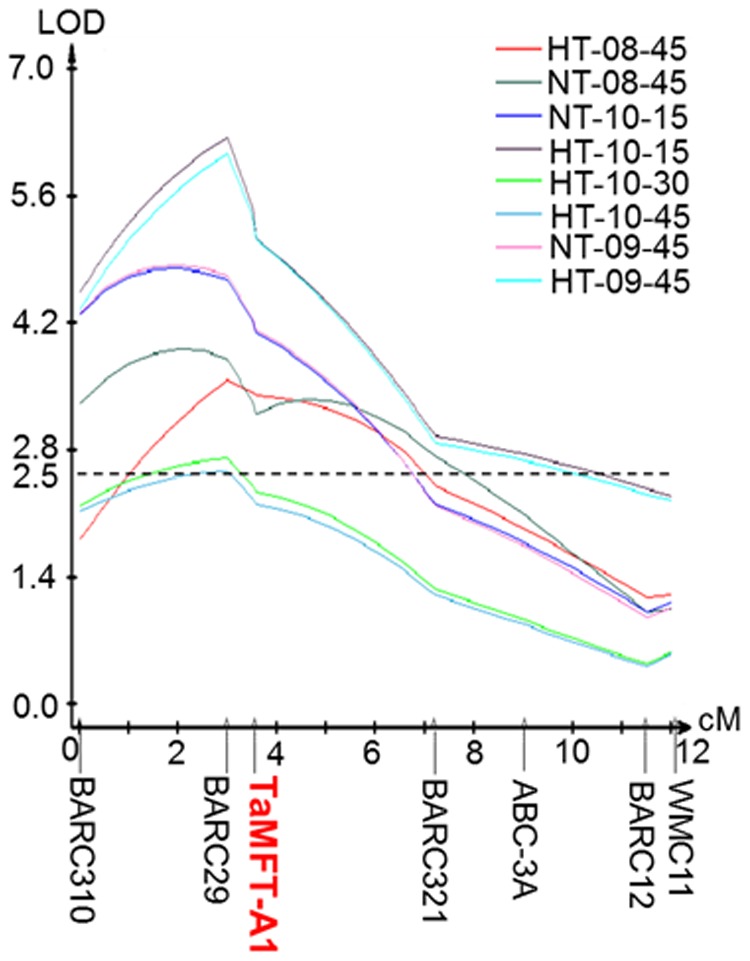
Chromosomal location and genetic effects of *QTsg.osu-3A* for seed germination. The QTLs were characterized at high temperature (HT) and normal temperature (NT) in years 2008, 2009, and 2010, when seed was harvested 15, 30, or 45 days. Germination rate was tested in the recombinant inbred lines (RILs) of the Jagger×2174 population. Molecular markers along the chromosome are placed as centimorgans on the horizontal axis. The horizontal dotted line represents a common threshold value of 2.5 LOD.

In order to find genomic regions causing the difference in seed germination between Jagger and 2174, the Jagger×2174 RILs were grown in field and their seeds were harvested and tested for seed germination at different temperature regimes (Table 1). The previously reported 350 SSR markers [24] were mapped in the population of Jagger×2174 RILs. When the phenotypes and genotypes were incorporated, a QTL for seed germination at high temperature and normal temperature was consistently mapped on the distal end of the short arm of chromosome 3A (Fig. 2), based on reference map of 5 SSR markers in hexaploid bread wheat [27]. This QTL for temperature sensitivity germination is thus referred to *QTsg.osu-3A*. This QTL did not have genetic effect on germination of seeds treated with low temperature (data not shown), as almost all seeds treated with low temperature germinated and no significant difference in seed germination rate among the RILs was observed. In order to confirm the physical location of *QTsg.osu-3A* on chromosome 3A, a gene encoding an ABC transporter that was known to locate on the short arm of chromosome 3A was also mapped at the *QTsg.osu-3A* locus (Fig. 2). *QTsg.osu-3A* spanned approximately 12 cM in genetic distance.

**Table 1:** A summary of genetic effects of *QTsg.osu-3A* on seed germination under various temperatures.

Year	Temperature (°C)	Days after harvesting	LOD	Phenotypic variation (%)
2008	25°C	45	3.9	19.3
	37°C	45	3.6	18.7
2009	25°C	45	4.8	22.5
	37°C	45	6.1	25.5
2010	25°C	15	6.2	22.5
	37°C	15	2.7	12.5
	37°C	30	2.6	11.8
	37°C	45	4.8	22.5

*QTsg.osu-3A* had LOD scores ranging from 2.6 to 6.2 accounted for 11.8–26.2% of the total phenotypic variation in seed germination tested at different years and different temperatures (Table 1). On average, *QTsg.osu-3A* explained 19.4% of the total phenotypic variation in the seed germination rate, indicating that *QTsg.osu-3A* had a significant and consistent genetic effect on seed germination in winter wheat.

### Allelic variation in *TaMFT-A1*

*TaMFT-A1* is a gene that is co-localized with *QPhs.ocs-3A.1*, a seed dormancy QTL mapped in spring wheat [2]. In order to test if *TaMFT-A1* is associated with *QTsg.osu-3A* mapped in this study, different primers specific to *TaMFT-A1* were designed, based on sequence alignment of *TaMFT-A1* with other two homoeologous genes, *TaMFT-B1* and *TaMFT-D1*. *TaMFT-B1* and *TaMFT-D1* sequences were derived from wheat genome sequence database (http://www.cerealsdblished.uk.net/) using the fragment sequences originated from Chinese Spring. Surprisingly, many primers that were expected specific to *TaMFT-A1* worked for 2174 but not for Jagger, suggesting that Jagger might have a greatly diversified *TaMFT-A1* gene.

**Figure 3:**
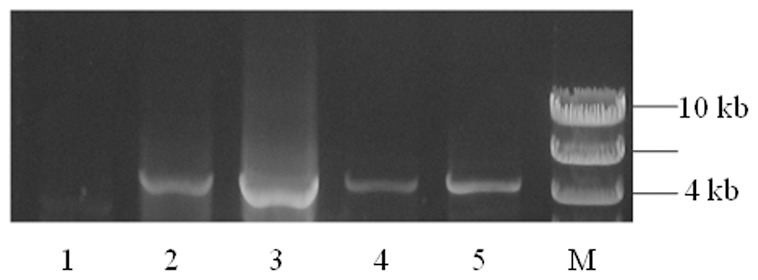
Specific amplification of *TaMFT-A1*. Primer MFT-F1M and MFT-A1R1 were used to amplify the complete *TaMFT-A1* from three nullisomic-tetrasomic (NT) Chinese Spring (CS) lines, N3AT3B (1), N3BT3A (2), N3DT3A (3), as well as Jagger (4) and 2174 (5).

**Figure 4:**
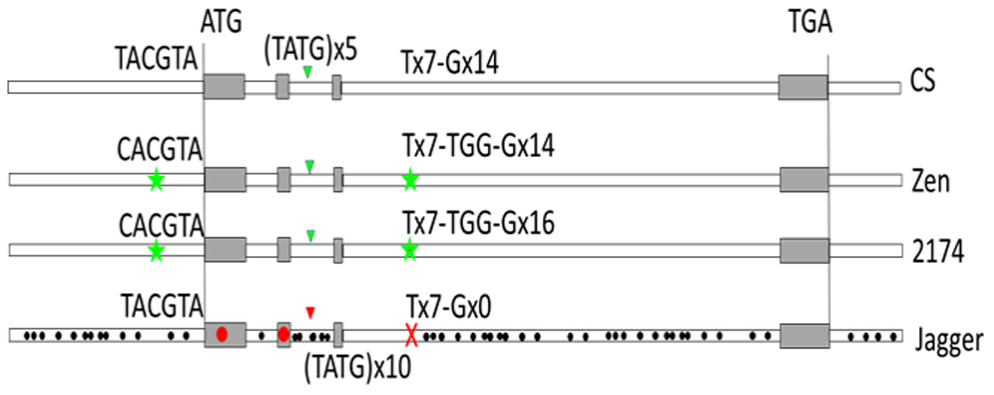
Diagram for mutated sites at the Jagger *TaMFT-A1* haplotype. Star symbol indicates positions of two reported polymorphisms in the CS allele for weak dormancy compared with the Zen allele for strong dormancy. Triangle star indicates the position of polymorphic site in intron 2 due to the presence of the poly ‘G’. Dot symbol indicates mutation sites throughout the Jagger *TaMFT-A1* gene compared with the other three alleles.

The complete gene of *TaMFT-A1* consisting of 4 exons and 3 introns from each of the Jagger and 2174 alleles was finally isolated using two primers which specificity to chromosome 3A was confirmed using Chinese Spring nulli-tetra lines of Chinese Spring (Fig. 3). The sequenced *TaMFT-A1* gene was 4,423 bp for the Jagger allele (GenBank accession: KF311059) and 4,330 bp for the 2174 allele (GenBank accession: KF311060). The Jagger *TaMFT-A1a* included 1,000 bp upstream from the start codon, 401 bp downstream from the stop codon, and 3,022 bp between the start codon and the stop codon; whereas the *TaMFT-A1b* allele included 1,012 bp upstream from the start codon, 401 bp downstream from the stop codon, and 2,917 bp between the start codon and the stop codon. The final sequence of the 2174 allele was exactly the same as AB571513 (Zen), except for a poly ‘G’ region, where 2174 had 2 more ‘G’ than Zen (Fig. 4).

Overall, there was 96% identity between the Jagger allele and 2174 allele. A total of 87 SNPs and 12 indels (insertions/deletions) with sequences from 1 to 20 bp were observed between the two alleles (Fig. 4). Two SNPs occurred in exons, one that occurred in exon 1 but did not cause alternation of amino acid, and the other that occurred in exon 2 and resulted in the alternation of one amino acid between Arginine residue in Jagger and Lysine residue in 2174.

**Figure 5:**
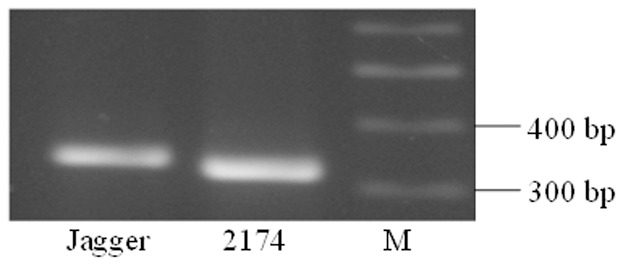
A PCR marker for *TaMFT-A1*. Primer MFT-A1F2 and MFT-A1R2 were used to amplify *TaMFT-A1* from Jagger (331 bp) and 2174 (319 bp). PCR products were directly run on a 1% agarose gel.

A PCR marker was developed to map *TaMFT-A1* in the Jagger×2174 RILs using primers MFT-A1F2 and MFT-A1F2. The amplified region included three indels, the first one showing 20 bp more in Jagger than 2174, the second one showing 8 bp less in Jagger than 2174, and the third one showing 15 bp less in Jagger than 2174. Without digestion, the PCR products from the two alleles were well separated in a 2% agrose gel (Fig. 5). *TaMFT-A1* was mapped under the center of *QTsg.osu-3A* found in the population of Jagger×2174 RILs (Fig. 2).

### The effect of *TaMFT-A1* on seed germination

**Figure 6:**
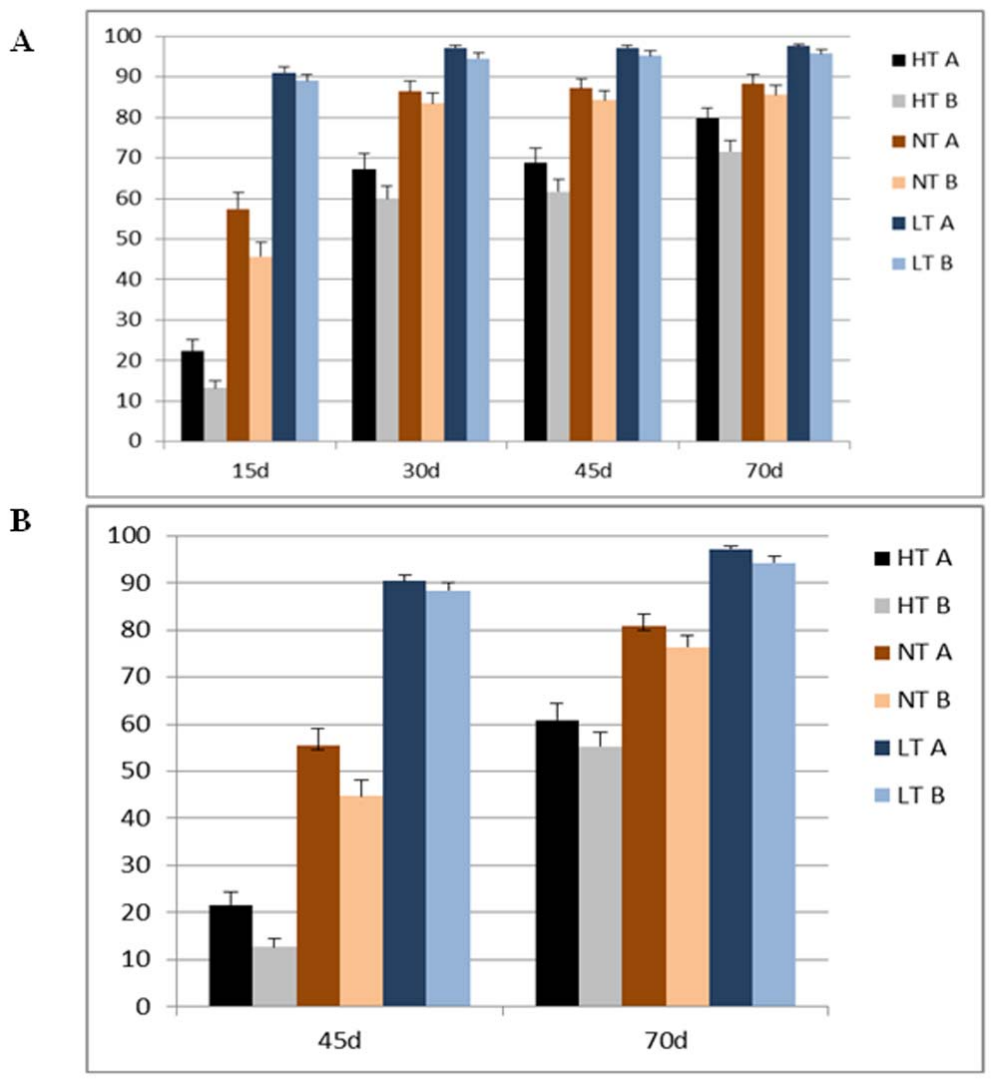
Genetic effect of *TaMFT-A1* on germination rate. The germination rate was averaged from each of the Jagger allele (A) or the 2174 allele (B) in the population (n = 96) that were characterized at high temperature (HT) and normal temperature (NT) in 2009 (Fig. 6A) and 2010 (Fig. 6B), when seed was harvested 15, 30, 45, or 70 days. Bar indicates standard error.

When the population was tested in 2009 (Fig. 6A) and 2010 (Fig. 6B), the averaged seed germination rate of the population was increased with the storage time. The trend was stable when seed was incubated at the normal temperature and the high temperature. At 15 days after harvesting, the averaged seed germination rate of the population was 17.8% at the high temperature, 51.6% at the normal temperature, and 90% after the seed was treated with the low temperature. These results indicated that the seed dormancy was broken by the low temperature, but the seed germination was inhibited by the high temperature, compared with the germination at the normal temperature.

The Jagger×2174 RILs were grouped into two types, one carrying the Jagger *TaMFT-A1a* allele and the other carrying the 2174 *TaMFT-A1b* allele. As shown in Fig. 6, *TaMFT-A1a* promoted seed germination, or *TaMFT-A1b* inhibited seed germination. Genetic effects of *TaMFT-A1* on seed germination were reflected at significant levels when the seed germination was tested at 15 days after harvesting at the normal temperature (p<0.05) and the high temperature (p<0.01) in 2009, and at 45 days after harvesting at the normal temperature (p<0.05) and the high temperature (p<0.01) in 2010. The germination rates at the other testing times were consistently higher in the lines carrying the Jagger *TaMFT-A1a* allele than the lines carrying the 2174 *TaMFT-A1b* allele, but the differences were not significant in statistical analyses (Fig. 6).

### Regulation of *TaMFT-A1* expression by temperature and seed storage time

**Figure 7:**
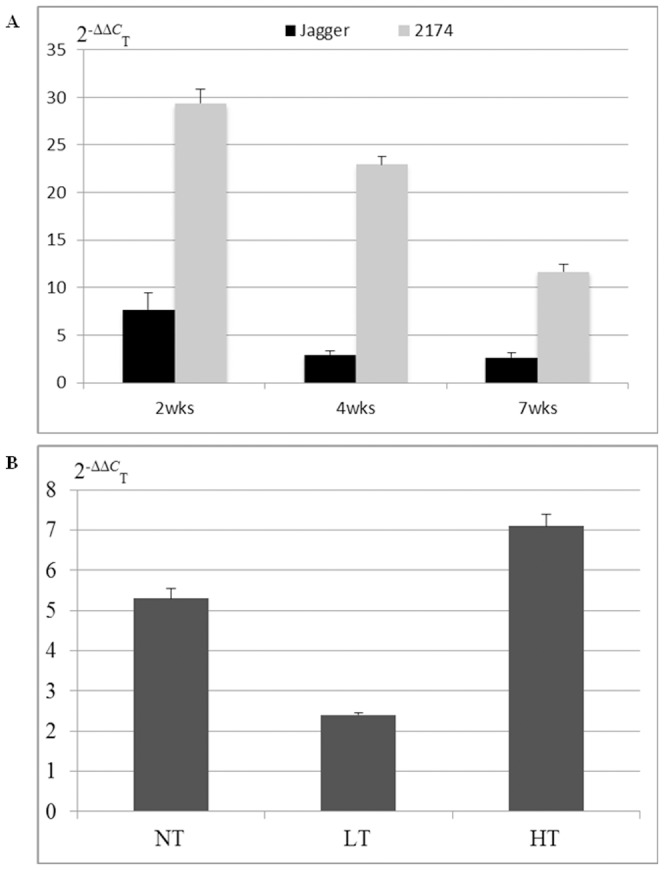
Expression profiles of *TaMFT-A1*. A) Transcript levels of *TaMFT-A1a* (the Jagger allele) and *TaMFT-A1b* (the 2174 allele) in the after-ripened seeds. RNA samples were collected from embryos of seeds, when the seeds were tested at 2 weeks (2 wks), 4 weeks (4 wks), and 7 weeks (7 wks) after harvest. B) Regulation of *TaMFT-A1* transcript levels by temperature. The RNA samples were collected from 2174 seeds that were treated with normal temperature (NT, 25°C), low temperature (LT, 4°C) for overnight, and high temperature (HT, 37°C) for 5 days. Transcript levels are shown using the values calculated by the 2^(−ΔΔCT)^ method, where CT is the threshold cycle, and *actin* was used as an endogenous control. The values represent mean expression levels (n = 12), and the bar indicates standard error.

Using RT-PCR, the *TaMFT-A1* transcript levels in germinated seeds of Jagger and 2174 at normal temperature (25°C) were determined (Fig. 7A). At 2 weeks after harvest, the Jagger *TaMFT-A1a* transcript level was 7.7, and the 2174 *TaMFT-A1b* transcript level was 29.5, which was approximately 3.8 folds of *TaMFT-A1a* in Jagger. The *TaMFT-A1a* transcript level was significantly decreased to 3 by 4 weeks and 2.7 by 7 weeks after harvest. The *TaMFT-A1b* transcript level was significantly decreased to 23 by 4 weeks and 11.7 by 7 weeks after harvest. These results indicated that the *TaMFT-A1* transcript levels at the normal temperature were down-regulated by the seed storage time.

In order to investigate how *TaMFT-A1* was regulated by temperature, the *TaMFT-A1* transcript levels in 2174 seeds treated with three temperature regimes were tested: i) continuous normal temperature (NT, 25°C), ii) low temperature (LT, 4°C), and iii) high temperature (HT, 37°C). As shown in Fig. 7B, the *TaMFT-A1* transcript level in germinated seeds treated with the low temperature was 2.4, which was 45.3% in seeds germinated at the normal temperature. The *TaMFT-A1* transcript level in germinated seeds treated with the high temperature was 7.1, which was increased 34% compared with the seeds germinated at the normal temperature.

### Diversity of *TaMFT-A1* among different ploidy wheat species

The primers MFT-A1F2 and MFT-A1R2 utilized for mapping were used to genotype cultivars/accessions of different ploidy wheat species. As summarized in Table S1, among 34 winter wheat varieties released in the southern Great Plains in recent years, one half of them were found to carry the Jagger *TaMFT-A1a* allele and the other half were found to carry the 2174 *TaMFT-A1b* allele. Among 19 pairs of parental lines that were used to construct mapping populations in the WheatCAP applied genomics project, 24 parental lines were found to carry the Jagger *TaMFT-A1a* allele and the remaining 14 parental lines were found to carry the 2174 *TaMFT-A1b* allele. Among 56 Chinese spring wheat cultivars/landrace, 20 were found to carry the Jagger *TaMFT-A1a* allele and the remaining 36 were found to carry the 2174 *TaMFT-A1b* allele. These results indicated that the Jagger *TaMFT-A1a* allele was not unique but was extensively utilized in many cultivars in different geographical areas.

The Jagger *TaMFT-A1a* allele is a novel haplotype, compared with the previously reported alleles, the Chinese Spring CS *TaMFT-A1* allele (AB571512) and the Zen *TaMFT-A1* (AB571513). In order to determine which allele is the wild type of *TaMFT-A1*, the primers MFT-A1F2 and MFT-A1R2 utilized for mapping were used to genotype diploid and tetraploid wheat species. Among 9 tetraploid wheat accessions of *T. turgidum* tested, only 1 accession showed the same allele as Jagger and the other 8 accessions showed the same allele as 2174, suggesting that *TaMFT-A1* has been diversified at the tetraploid level. The complete sequence of *MFT-A1* from *T. turgidum* ssp. *durum* that was used to construct BAC library [28], the *MFT-A1* allele from the tetraploid wheat (GenBank accession: KF311061) showed 96% identity and 113 SNPs or indels compared to the Jagger *TaMFT-A1a* allele and 98% identity and 50 SNPs or indels compared to the 2174 *TaMFT-A1b* allele.

All of 3 diploid wheat accessions of *T. urartu* tested showed the same allele as Jagger. However, a section of 844 bp including partial exon 1, exons 2 and 3 from the *MFT-A1* of *T. urartu* (PI 428183) (GenBank accession: KF311062) was 94% identity to the Jagger *TaMFT-A1a*, 97% to the 2174 *TaMFT-A1b*, and 97% to the *T. durum MFT-A1*. The *MFT-A1* of *T. urartu* showed multiple SNPs or indels with the homologous genes in tetraploid and hexaploid wheat, suggesting that the *MFT-A1* gene has greatly diverged during the evolution from diploid through tetraploid to hexaploid wheat.

## Discussion

Previous studies on seed dormancy and germination in wheat have been focused on pre-harvest sprouting in spring wheat. More than 15 genetic loci have been reported that are responsible for this critical trait in a large range of wheat areas worldwide, including *QPhs.ocs-3A.1* that was repeatedly mapped on the short arm of chromosome 3A using RILs derived from a cross between the highly dormant wheat cultivar Zenkoujikomugi (Zen) and the less dormant cultivar Chinese Spring (CS) [2], [18], [29], [30]. *TaMFT-A1* was mapped co-segregated with a SSR marker *Xbarc310* on the short arm of chromosome 3A in an F_2_ population derived from a cross between CS and CS (Zen3A) [2]. In this study, we mapped *QTsg.osu-3A* for seed germination in the population that was generated from a cross between two winter wheat cultivars and found that *QTsg.osu-3A* was tightly associated with *TaMFT-A1*. Liu et al. [1] also found that *Xbarc310* was associated with a major QTL for pre-harvest sprouting in the US hard white winter (HWW) wheat cultivar ‘Rio Blanco’. These studies have pointed to *TaMFT-A1* which may play an important role in moderating seed dormancy/germination under various temperature conditions in spring and winter wheat cultivars. This may provide an opportunity to compromise the efforts of *TaMFT-A1* on seed dormancy, pre-harvest sprouting, and seed germination in wheat breeding.

One of the initial aims in this study was to explore the genetic loci involved in controlling high temperature germination sensitivity. The results indicated that differential germination ability under high temperature between Jagger and 2174 was partly due to the *TaMFT-A1* locus. The dormancy rescues in the after-ripened seeds of winter wheat cultivars could be immediately released by low temperature, gradually released with storage time at normal temperature, and maintained at high temperature. The down-regulation of the *TaMFT-A1* transcript level by low temperature and up-regulation of *TaMFT-A1* transcript level by high temperature were consistent with the effects of the temperatures on seed germination in winter wheat. This study suggested that *TaMFT-A1* played an important role in the variation in seed germination in winter wheat cultivars when they are planted earlier to produce more biomass to graze the cattle in the southern Great Plains.

The *TaMFT-A1* gene promotes or maintains seed dormancy in embryos matured at lower temperature (13°C) in comparison to favorable temperature (25°C) in wild type wheat, which is confirmed by the overexpression of *TaMFT-A1* preventing germination in transgenic wheat [2]. The previous study indicated a contrasting effect of the lower temperature on *TaMFT-A1* with this study that showed that *TaMFT-A1* was down-regulated by low temperature. This difference is probably due to the seed state difference (seeds before physiological maturity vs. after-ripened seeds). Low temperature is usually used to promote seed germination in winter wheat research and production, though a mechanism involving this alternation is unknown. This study indicated that *TaMFT-A1* is a repressor in germination of the after-ripened seeds in winter wheat, and the repressor in the after-ripened seeds can be removed by low temperature.

Two polymorphic sites in *TaMFT-A1* exist between the CS allele for weak dormancy and the Zen allele for strong dormancy. One SNP is located 222 bp upstream from the initiation codon of *TaMFT-A1*, which is believed to be a cause of differential expressions due to the presence of a substitution of T in non-dormant CS with a C in the dormant Zen cultivar that occurs in the A-box motif, a bZIP transcription factor binding site [2]. The 2174 allele for the low seed germination was exactly the same sequence in the promoter as the Zen allele for the high seed dormancy, supporting that the polymorphic site is important in controlling *TaMFT-A1* expression. However, the Jagger *TaMFT-A1* allele that was found in this study for the high germination of the after-ripened seeds is very different in the sequence from the CS *TaMFT-A1* allele that was identified for the low dormancy of the immature seeds. It is not known if the Jagger *TaMFT-A1* allele and the CS *TaMFT-A1* allele have different mechanisms in regulating seed dormancy and germination. Winter wheat cultivars have fewer problems with pre-harvest sprouting than spring wheat cultivars [1]. No pre-harvest sprouting was observed in Jagger or other winter wheat cultivars derived from pedigrees of Jagger in nurseries for years [11]. The Jagger *TaMFT-A1* allele in winter wheat can be used to replace the CS *TaMFT-A1* allele in a spring wheat cultivar to test if pre-harvest sprouting can be avoided in new spring cultivars.

*MFT* is a homologue of the phosphatidylethanolamine-binding proteins (PEBP) *FT* and *TFL1*, which have opposite roles in the promotion of flowering [31], [32], [33]. *FT1* on chromosome 7B in the temperate crops showed the most robust induction of flowering under long days in wheat [34]. *FT-D* on chromosome 7D ( = *VRN-D3*) was found to affect physiological maturity in the same population as *TaMFT-A1* [35]. However, no significant genetic effect was observed for the *FT-D* gene to affect seed germination or for *TaMFT-A1* to affect physiological maturity, suggesting that the two members of the *FT* gene family have functionally diversified during the evolution of wheat.

The previous study suggested that the variation between CS and Zen was mutated during domestication of common wheat [2]. However, the substantial variation between the 2174 *TaMFT-A1b* allele and the Jagger *TaMFT-A1a* allele suggested that the two *TaMFT-A1* alleles should be originated from different *TaMFT-A1* genes at tetraploid wheat. It has been repeatedly reported that common wheat cultivars have different tetraploid wheat donors [36]. The diversity of *TaMFT-A1* among different wheat species suggested the presence of multiple *TaMFT-A1* alleles in hexaploid wheat, and further functional identification of each allele in hexaploid wheat is needed for the utilization of *TaMFT-A1* in wheat molecular breeding.

## Supporting Information

Table S1 **Wheat cultivars/accessions used for determining the frequency of the** ***TaMFT-A1*** **alleles.**
(DOCX)
