# Supplementary material for: TaMFT-A1 Is Associated with Seed Germination Sensitive to Temperature in Winter Wheat
Source: PLoS One. 2013 Sep 12;8(9):e73330. doi: 10.1371/journal.pone.0073330 (PMC3772017; doi:10.1371/journal.pone.0073330)
Supplement: Table S1 — Wheat cultivars/accessions used for determining the frequency of the TaMFT-A1 alleles. (DOCX) [file pone.0073330.s001.docx]

**Table S1** **Wheat cultivars/accessions used for determining the frequency of the *TaMFT-A1* alleles**

| Allele | Source | Cultivar/line/landrace/accession |
| --- | --- | --- |
| Jagger allele | 2X wheat  4X wheat  Chinese cultivars  CAP  SGP | *T. urartu* ( PI 428183, PI 428180, PI428323)  *Turgidum* (PI 352541)  Yang9663, Huaimai19, Yannong578, Xinmai19, Ning0088, Ningmai11, Wanmai33, Hao9409, Yangmai16, Yangmai18, Xinmai18, Yanfu188, Suxu3, Lianmai1, Pin14, Yannong19, Jinan13, Baifengmai, Yannong21, Zhengmai004  UC1110 (CA), CIMMYT-2 (PI 610750) (CA), IDO444 (ID), Zak (ID), Stephens (OR), OR9900553 (OR), Finch (WA), Eltan (WA), Louise (WA), Panawawa (WA), GRN*5/ND614-A (MN), NY18/ Clark’s Cream 40-1 (MN),  Platte (CO), CO940610 (CO), Jagger (OK, NE), Harry(KS), Heyne (KS), Wesley (KS), Weebill (TX), Pio 25R26 and Foster (NY), Cayuga (NY), USG3209 (VA).  Fuller, Jagger, OK Bullet, Santa Fe, Shocker, Endurance, Doans, Deliver, Jei110, Guymon, Lakin, TAM112, Neosho, Duster, Protection, Endurance, Ripper |
| 2174 allele | 4X wheat  Chinese cultivars  CAP  SGP | *T. durum* (PI 366990, PI 191654, PI 384392), *T. turgidum* (PI 347135, PI 113393, PI14082), *T. dicoccum* (PI265004, PI 286061).  Sushenmai1, Yang9817, SuB, Yangmai158, Wanmai147, Yangmai11, Yang0188, Huamai0027, Yang05-334, Yang06-311, Yangmai7, Taikong5, Yangmai19, Yangmai17, Yangfumai1, Sushenmai1, Yangmai158, Yang06-135, Yang06-615, Yang05-311, Yang03-77, Yang06-385, Guandong107, Yang0135, Gaoyou503, Yangmai12, Hu438-15, Yangfymai2, Baiyingdong2, Baihuomai, Heixiaomai, Zixiaomai, Zheng99081, Wanmai18, Yangfu0082, Xumai26.  Rio Blanco (ID), ID0556 (ID), McNeal (MT), Thatcher (MT), TAM105 (NE), KS01HW163-4 (KS), 2174 (OK), Jupeteco (TX), SS550 (GA), PIONEER 26R46 (GA), P91193 (IN), P92201 (IN), Cayuga (NY), Jaypee (VA).  OKfield, Centerfield, Overleg, Jagalene, 2174, TAM111, Cutter, Fannin, Danby, Trego, OK102, TAM110, Above, Custer, Hatcher, Intrada, Overleg. |

CAP: Coordinated Agriculture Project. SGP: Southern Great Plains.
